# Network causality, axonal computations, and Poffenberger

**DOI:** 10.1007/s00221-017-4948-x

**Published:** 2017-05-09

**Authors:** Giorgio M. Innocenti

**Affiliations:** 1 Department of Neuroscience, Karolinska Institute, Stockholm, Sweden; 2 Brain and Mind Institute, and Signal Processing Laboratory, École Polytechnique Fédérale de Lausanne, Lausanne, Switzerland

**Keywords:** Neural networks, Axonal morphology, Transmission delays, Corpus callosum, Cortico-striatal connections

## Abstract

**A**ll brain operations are implemented by networks of neurons. Unfortunately, the networks underlying even the most elementary brain operations remain elusive. This is due to the complexity of the networks, their heterogeneity, and to the multiple computations performed by the axons. Poffenberger’s paradigm is one example of a simple task aimed at characterizing the temporal properties of an interhemispheric network which has remained elusive to this day.

## Introduction

It is an honor and a pleasure to contribute to the 50th anniversary of Experimental Brain Research, co-founded by my, early, and too early disappeared post-doctoral supervisor Otto Creutzfeldt, and where I published some of my papers long before being coopted to Editorial Board member.

And time forces me to face the question: what did I learn over the last 50 years? Or rather did I understand anything worth talking about? I will try to summarize one aspect of neuroscience which unfortunately seems to take us to the limits of what can be understood.

## Network causality

**Fig. 1:**
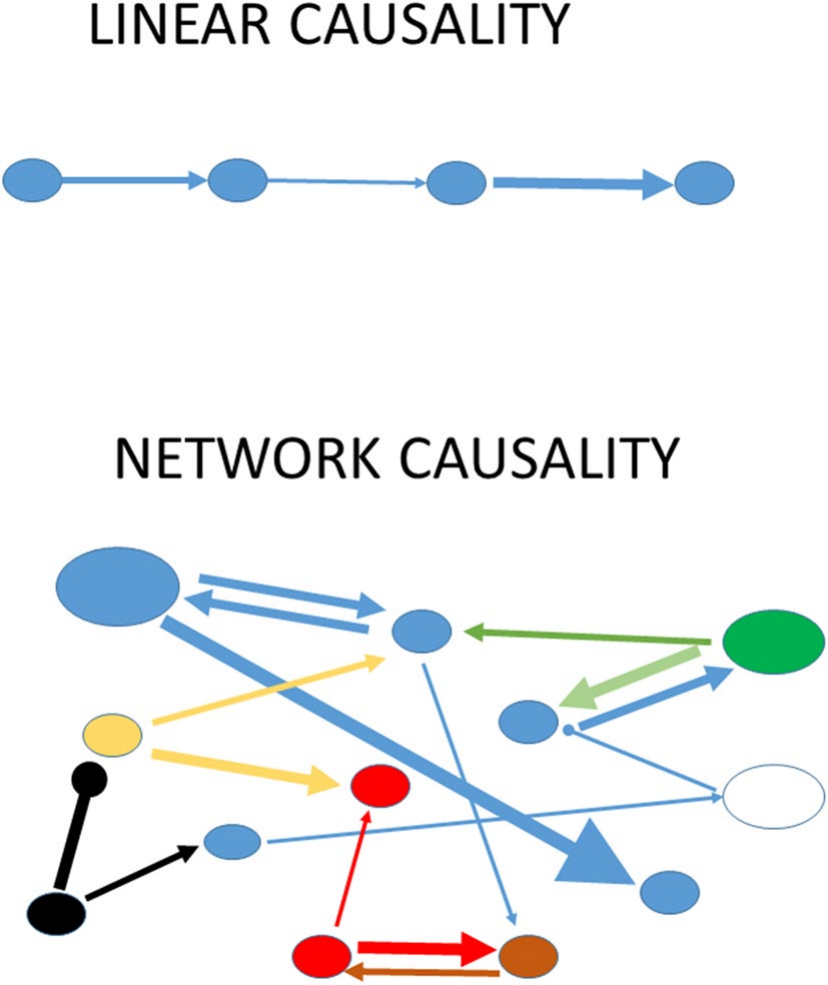
Linear causality and network causality, schematized. The following conventions are included as they apply to neural networks. Ovals represent neurons or neuronal pools of different sizes and genetic/epigenetic makeups (*colors*). *Arrows* denote connections between neurons or neuronal pools, their direction information flow, *colors* denote heterogeneity in neurotransmitter and/or co-transmitters. *Thickness* and *length* of *arrows* denote the speed of axonal conduction and conduction distances (as in Fig. 5), which, together, determine conduction delays (not shown). The type of ending denotes excitatory (*arrowheads*) or inhibitory (*circles*) connections, their size, the size of synaptic boutons, and indirectly the strength of the connection (Innocenti and Caminiti 2016). The diagram of linear causality exaggerates homogeneity while that of network causality stresses the heterogeneity of neural connections

The concept of causality is at the roots of all sciences, including brain sciences. One can distinguish between two types of causality: linear causality and network causality (Fig. 1). Linear causality is characterized by one agent affecting one object, which in turn can affect another object and so on. This kind of causality is easily described by elementary mathematics, as in physics when one force causes the acceleration of a mass, which can in turn cause the acceleration of another mass etc.

Network causality applies to situations where one agent can affect multiple objects, each of which can affect other objects which in turn can feed-back on each other etc. This kind of causality is more difficult to describe by simple mathematical formulations, although each of the interactions among its elements can. One can readily recognize that network causality applies to most phenomena in the field of biology, economics, or social sciences. I have previously argued that network causality describes the interactions occurring among elements involved in the development of the nervous system, from genes to phenotype (Innocenti 1991).

Network causality typically applies to the networks of neurons which implement brain operations. I will refer to biological networks of neurons as NONs, while neural networks (NNs) denotes networks of artificial neurons which mimic NONs.

The central goal of the neurosciences is to map states of the “mind” onto states of the brain (Innocenti 1993), i.e., essentially on the activity of NONs. It is not obvious that we have the mathematical tools to tackle network causality rigorously, in its most complicated and unfortunately most common occurrences. Tools have been developed, though, they include Bayesian networks, direct acyclic graphs, and Ishikawa diagrams. The question is if they are sufficient to deal with neural networks.

In the case of the brain, difficulties arise from at least five features of NONs. First, the elements of the net, were they neurons or groups of neurons, are heterogeneous. Second, the strength of the interactions differs among the elements of the network. Third, the interactions among elements of the network might be non-linear. Fourth, some of the interactions might be of the Boolean AND type and others of the OR type. Finally, the interactions occur in a temporal frame, whereby some interactions can be faster, while other might be slower (Fig. 1).

Trying to be pragmatic one can ask: what do we really want to know about neural networks? The activity of the individual neurons provides windows on the activity of the network, but we are unable to characterize in real time the activity of all the neurons in the net, not even that of the most significant ones, i.e., those whose disappearance or malfunction would severely disturb the output of the network.

A second question is: can we bypass the full knowledge of the dynamics of all the elements of the network by recording their collective activity? Indeed, we have accumulated significant knowledge by recording the activation of brain sites with methods temporally and/or spatially accurate but not both, such as cross correlations of single neuron activity, of fMRI and EEG signals or the more recent extensions including local field potential, voltage sensitive dyes, or calcium imaging.

It seem plausible that a fruitful avenue to grasp the dynamics of NONs in behavioral or cognitive states shall come from massive simulations of neuromorphic networks. Still, one fundamental question is: which features of the NONs should be entered in the simulations? This paper focusses on the axon which is the key connector (an edge, in graph theory), whereby neurons can modify each other’s activity.

## The computational properties of axons

Axons display a wide variety of morphologies, some of which were already described by Ramon y Cajal, using Golgi’s silver impregnation method, others were revealed by axonal transport of tracers and serial reconstruction of axonal terminal arbors (for examples, see Fig. 2 in Innocenti 2011; Massé et al. 2016; Mengual et al. 2016; Parent and Parent 2016).

**Fig. 2:**
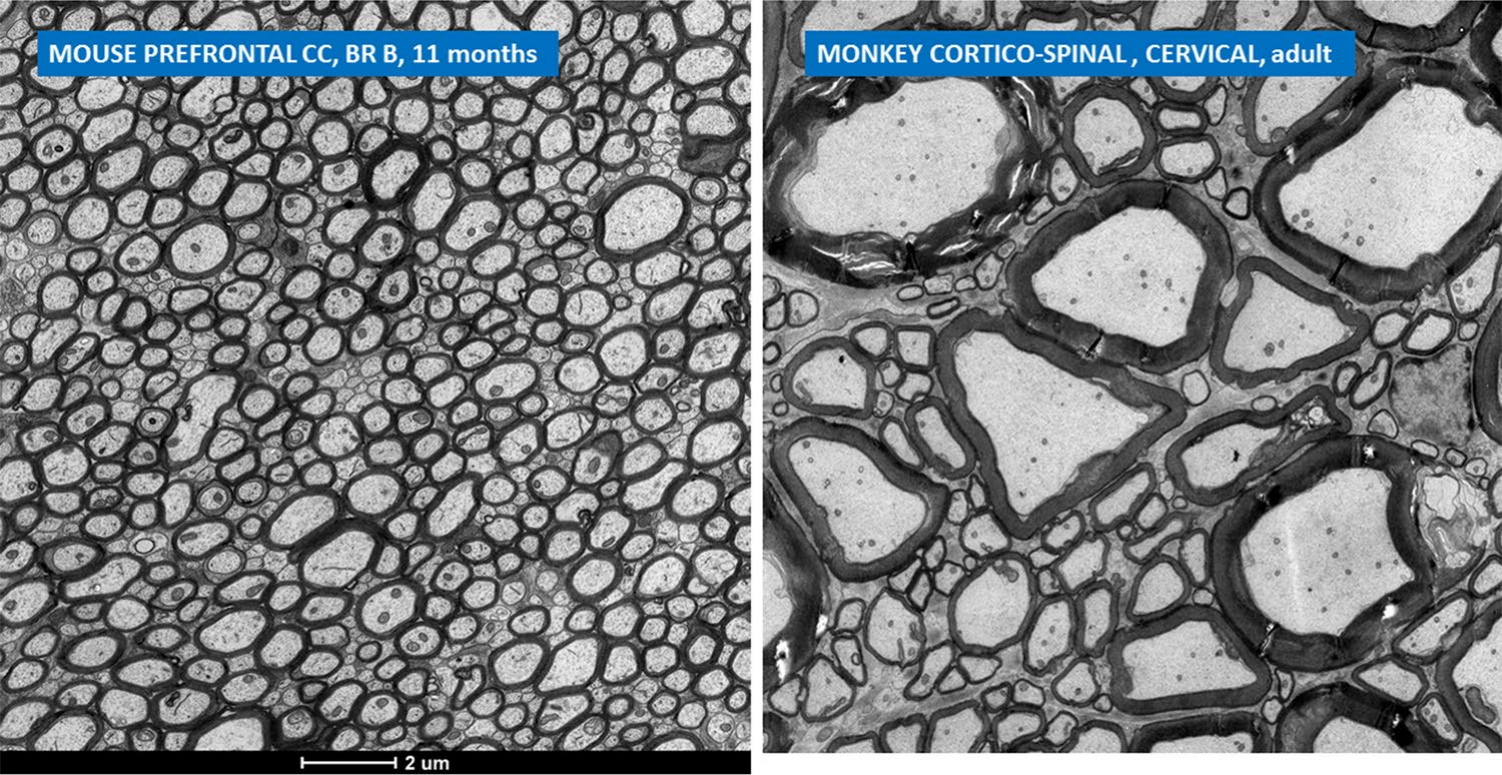
Electron-microscopic images of the genu of the CC in a mouse in the sector, where prefrontal axons are crossing (*left*) and of the lateral funiculus of the spinal cord of the macaque monkey, at the cervical level, corresponding to the sector containing cortico-spinal axons (*right*). Notice the disappearance of unmyelinated axons and the appearance of very large axons in the monkey. Unpublished

In spite of the differences, several aspects of axonal morphology are conserved across species and systems. In comparing axonal arbors as different as the thalamo-cortical axons to the barrel field in two strains of mice and the callosal axons to visual areas in the cat (Tettoni et al. 1998), we found that the following parameters were conserved: total arbor length, number of branches, maximal order of branching, branching angles, topological distribution of branches, and topological distribution of boutons (for an extended analysis of boutons’ distribution in cat visual cortex, see Anderson et al. 2002). What differed was the size of the conduction compartment (the sector of the axon conducting the action potential but devoid of synaptic boutons) and of the transmission compartment (the sector of the axon carrying synaptic boutons). In addition, the diameter of the axons varies enormously across axons, mainly in the conduction compartment. Compare, for example, axons in the prefrontal sector of the corpus callosum of the mouse, with axons in the cortico-spinal sector of the monkey lateral funiculus (Fig. 2).

**Fig. 3:**
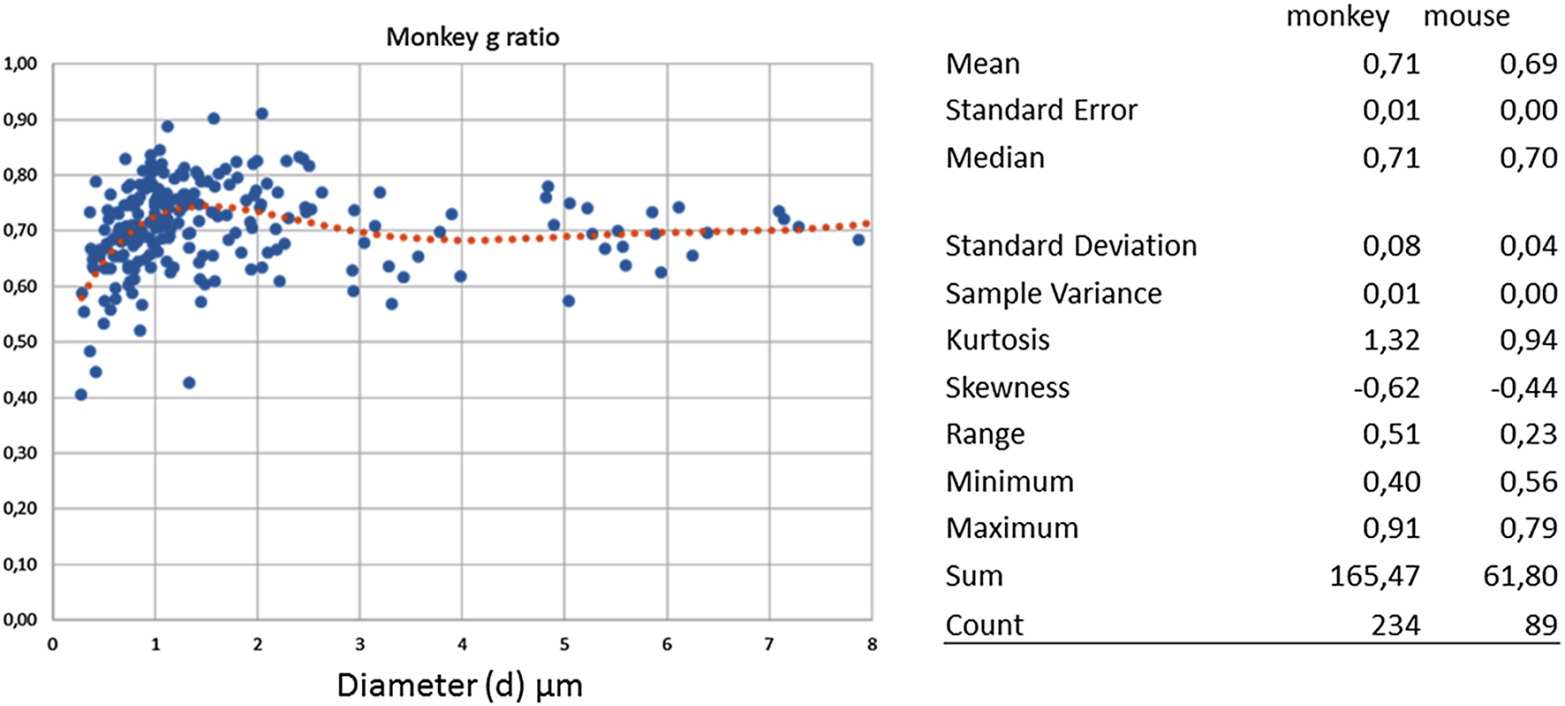
The ratio between the inner axonal diameter (*d*) and the outer diameter (*D*): *g* = *d*/*D* in the lateral funiculus of the spinal cord and in the genu of the CC of the mouse (above). Notice that on average the *g* ratio is constant across species and for different axon diameters, although with a scatter, whose functional consequences are unknown. Unpublished

Differences are due to the disappearance of unmyelinated axons in the monkey, as well as to the increased diameters of a fraction of myelinated axons. In spite of these differences, another quasi-constant axonal parameter is the ratio (*g*) between the inner—(*d*) and the outer—(*D*) diameter of the axon the *g* ratio *g* = *d*/*D*. As predicted by theoretical work of the 70s, this parameter is almost identical in the two sets of axons, being 0.71 in the monkey and 0.68–0.69 in the mouse (Fig. 3). This is important, since the g ratio, together with axon diameter (*d*), determines axonal conduction velocity *V*:

$$V=\left( {5.5/g} \right) \times d$$

i.e., a substantial part of the temporal computations axons perform, as further elaborated below. The third parameter, the distance between Ranvier nodes (where action potential is conducted in a saltatory way), is also believed to increase linearly with axon diameter although with considerable scatter (Ibrahim et al. 1995).

Morphological similarities and differences across axons underlie common functional roles. I have repeatedly suggested that the axons, unlike cables in an electronic circuit, do not faithfully convey information between neurons, but instead participate in signal processing in at least three domains: mapping, amplification, and timing (Innocenti et al. 2016a and references therein). Mapping involves transforming the topographical position of the cell body of origin into the position of its terminal boutons. Amplification involves distributing different numbers of boutons (as well as boutons of different sizes; below) to different targets. Temporal transformations are implemented by reaching different targets with different delays, due to the conduction properties of the branches of each axon and to the different conduction properties of different axons. It was also suggested that axons of different diameters may conduct different frequencies of action potentials (Perge et al. 2012). Further computations include (1) activity dependent shaping of action potential; (2) signal amplification along the axons; and (3) axonal integration (Debanne 2011). More complex scenarios include probably rare events such as the possibility of failure or delays in spike propagation at branching points, delays caused by varicosities etc (see Massé et al. 2016 for data on visual areas connections in the mouse and comprehensive discussion).

**Fig. 4:**
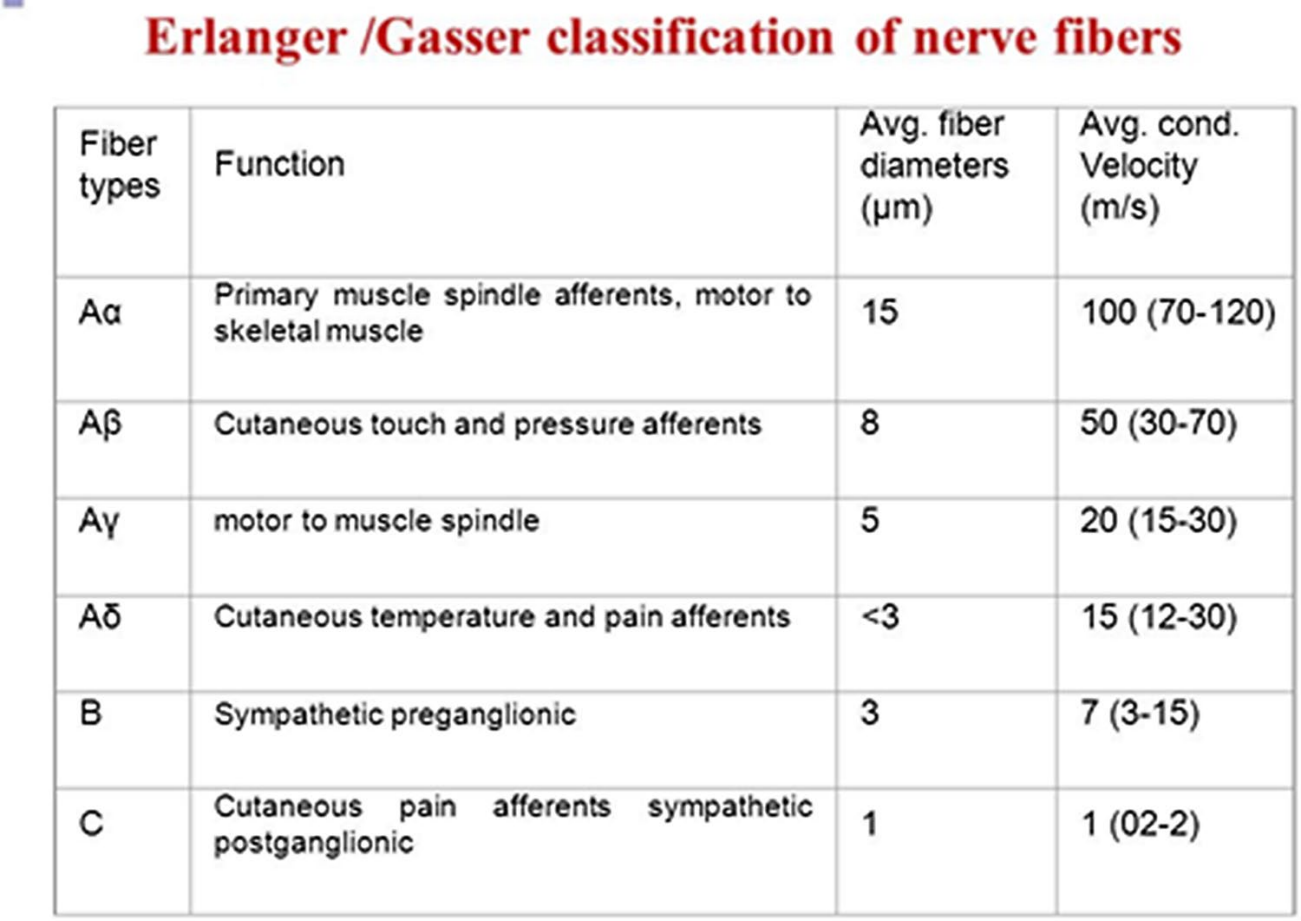
Erlanger and Gasser’s classification mapping functional properties on peripheral axons of different diameters and conduction velocities. Available at: https://goo.gl/images/F7y61c

Among the various computations performed by axons, the temporal transformations implemented by axons of different diameters have a long and distinguished history in the peripheral nervous system. Indeed, Erlanger and Gassers classification, directly related fiber diameter, to conduction velocity and to functional properties (Fig. 4). Those findings were awarded Nobel Prize in Physiology or Medicine in 1944.

**Fig. 5:**
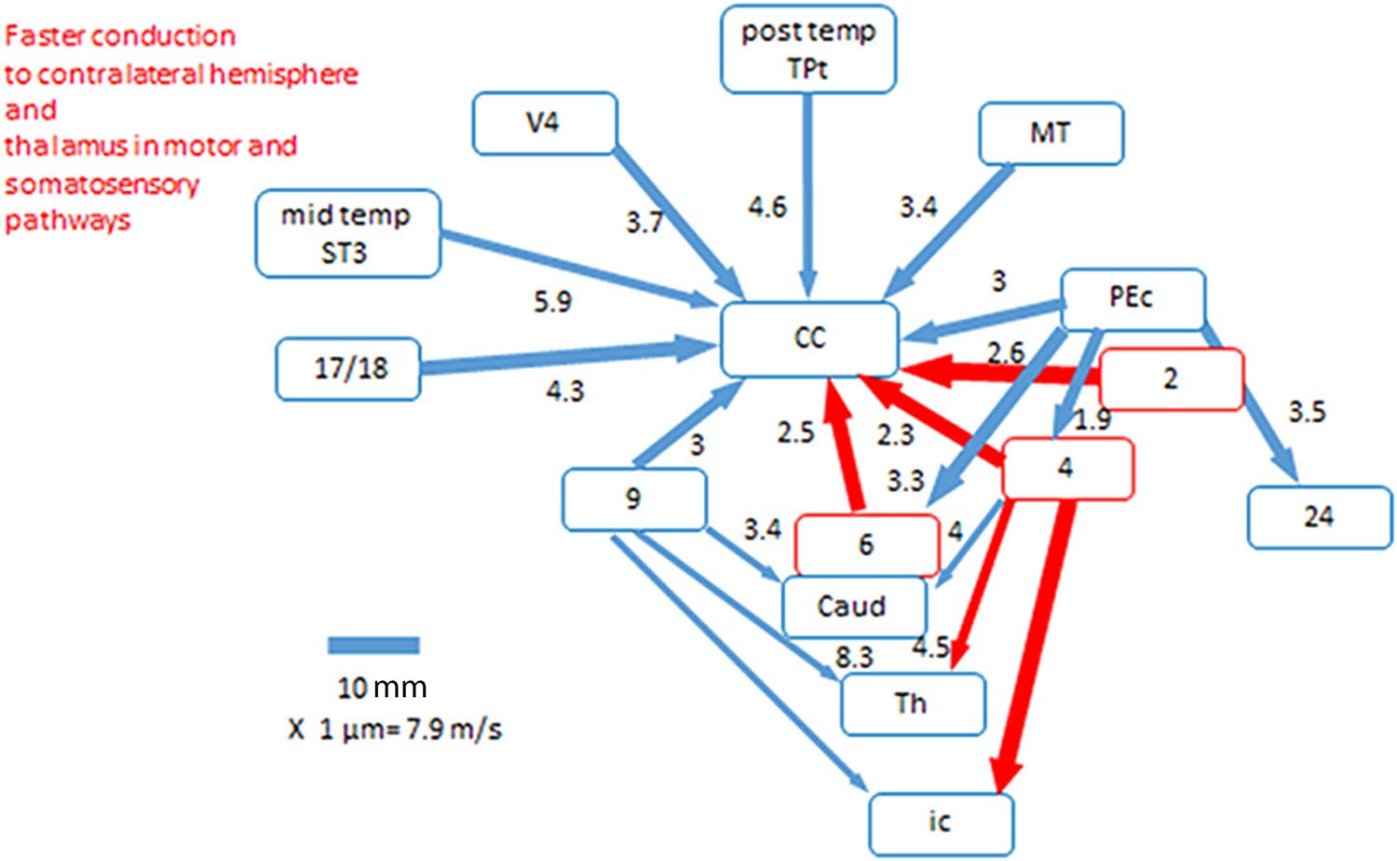
Schematic representation of diameters and estimated conduction delays for connections traced in Tomasi et al. (2012) and Innocenti et al. (2014). *Thickness of arrow* segments is proportional to median diameter; their length is proportional to pathway length; and projections from area 4 are the means of two animals. Delays are calculated on conduction velocities and pathway lengths. *Red arrows* emphasize the faster conduction of pathways originating in somatosensory and motor areas. Reproduced from Innocenti et al. (2016a). Main acronyms (Paxinos et al. 2000): *ST3* superior temporal area 3, *TPt* temporoparietal area, *PEc* parietal area caudal, *MT* middle temporal area (visual area5), *Caud* N caudatus; *Th* thalamus, *ic* internal capsule, *CC* corpus callosum. Numbers correspond to Brodman areas

Axon diameter differences in the central nervous system have come into focus recently, since the discovery that different cortical connections use axons of different diameters (Caminiti et al. 2009). The finding was heralded by the report that different sectors of the primate corpus callosum (CC) contain axons of different diameters (Aboitiz et al. 1992; LaMantia and; Rakic 1990). Since different CC sectors also contain axons of different cortical origins, the inference that different areas may communicate with different diameter axons was rather attractive. Indeed, that was validated with injections of anterograde tracers in different areas in the monkey (Caminiti et al. 2009; Tomasi et al. 2012). This revealed a hierarchy with thinner CC axons originating from prefrontal, parietal, and temporal association areas and thicker axons originating from primary motor, somatosensory, and visual areas. From the diameter of the axons, their conduction velocity could be computed and from conduction velocity and pathway length, one could calculate the delays generated by the axons from their cortical origin to the midline of the CC and to the contralateral cortex. This established a hierarchy predicting faster conduction from premotor, motor, and somatosensory areas and slower conduction from the temporal areas. Obviously, similar concepts could be extended to other cortical connections and this was done by considering short intracortical connections, cortical connections to the thalamus, to the basal ganglia as well as connections from parietal cortex to frontal and prefrontal areas (Innocenti et al. 2014). These findings are presented in Fig. 5 where the different axonal systems are characterized by their median axon diameter, pathway length, and conduction delays.

The general messages which can be gathered from those data are that: (1) axonal diameters depend not only on the area of origin of a projection but also on the target; that is, the same area will send axons of different diameters to its different targets. For example, prefrontal area 9 will send thicker axons to the CC, thinner ones to the striatum, thalamus, or internal capsule. (2) Unlike the long connections, the short intracortical connections seem to be similar across areas. (3) The axon diameter of the projections and the pathway lengths can be expected to generate shorter conduction delays for the premotor, motor, and somatosensory connections. We have speculated that the latter finding might indicate that motor and somatosensory areas process more information per unit time and that this could establish a reference baseline, or temporal frame, for the information pertaining to other sensory modalities, or resulting from operations of intracortical association. More specifically, we have proposed that the higher motor and somatosensory processing speed may be an important component of the mechanisms responsible for the sense of body ownership (Innocenti et al. 2014).

One important missing information is whether faster and slower axons also differ in other properties as discussed elsewhere (Innocenti 2017), namely, their targets: First, neurons required to respond and drive targets fastest might receive input from fastest axons as this is the case for the magnocellular and parvocellular retino, geniculate, cortical projections. Second, in some cases, the inhibitory interneurons seem to receive the fastest input as we reported for the visual callosal connections of the ferret (Makarov et al. 2008).

The acquisition of axon diameters in the adult brain is the combined outcome of development and evolution, operating in very different time frames from million years to months (Innocenti 2011). Two processes proceed in parallel: (1) an increase in the number of myelinated axons and (2) the appearance of a cohort of thicker axons. These are two aspects of neuronal differentiation which, together with the enlargement of the brain, lead to an increased spectrum of conduction velocities and of conduction delays whose consequences can be investigated with the help of computational models (Caminiti et al. 2009; Innocenti et al. 2016a, for review).

Several lines of work suggest that axon diameter might be regulated by environmental factors in development and that environmental control might continue in adult life (see Innocenti et al. 2016a, for review).

It is important to realize that axon diameter correlates with other aspects of neuronal morphology, both of which may have computational consequences. The diameter of cortical axons appears to correlate with the size of the parent cell body, as it happens in other CNS structures, notably the motor neurons and the retino-geniculate projection. It is not clear which additional properties the large cell body confers to interneuronal communication in the cortex. In the case of motor neurons, the size of the cell body relates to the threshold for activation (the so-called Henneman principle). The possibility that this might apply to cortical neurons as well was tested by Fromm and Evarts (1981), but the expectation was not robustly confirmed.

The diameter of an axon also correlates with the size of the synaptic boutons it delivers and the latter might correlate with the amount of neurotransmitter released (Innocenti and Caminiti 2016). Therefore, the size of synaptic boutons, not only their number, participates in the processes of signal amplification implemented by axons at their site of termination. Unfortunately, we do not know if axons of different diameter also reach different targets, i.e., different types of neurons or different dendritic or somatic compartments of a neuron.

Indeed, what was outlined above provides a very rough and incomplete picture of the complexity of causal interactions in CNS neuronal networks, focused on the role played by the axons. The other key player in this interaction is of course the dendritic arbor which shows different temporal and amplification properties at different locations (Branco and Häusser 2011).

## Networks of neurons and behavior: Poffenberger’s paradigm

The complexities of neuronal interactions sketched above seem to discourage the hope of describing behavior and cognition in terms of causal NONs. Nevertheless, this might be possible in some cases.

One approach is to choose NONs which appear to be particularly simple. This view probably boosted the tremendous success of visual system studies over the second half of last century. The visual pathways from the retina through the Lateral Geniculate Nucleus to the primary visual cortex are indeed relatively simple. In addition, the retinofugal projection can be dissected into the anatomical and physiological properties of its constituent neurons, in particular, those of the magnocellular and the parvocellular pathways. Studies of the visual system profoundly influenced the psychology of perception and led to the first implementations of man-made perceptual devices. Thanks to Marr’s theoretical approach (1982), they also clarified the path between brain and “mind” by identifying three levels (computational, representational/algorithmic, and implementation) in this path. Still, the project of mapping vision on NONs is incomplete and fragmented beyond the primary visual cortex.

Another approach is to choose a simple behavior, in a rather uncomplicated nervous system, to characterize and model the physiological and morphological elements of the NONs; characterization and modeling are progressively refined by sequential interactive approximations. This approach was successful, for example, in the description of the NONs responsible for the control of body movement in the lamprey (Grillner et al. 2007).

A third approach is to choose networks of neurons interconnected by short axons (microcircuits or local circuits), whereby the contribution of the axons can be compounded in the measured interactions between morphologically and electrophysiologically identified neurons. This very laborious approach has provided interesting results for the cerebral cortex albeit far from revealing what the defined NON might be computing (Markram et al. 2015; Jiang et al. 2016).

**Fig. 6:**
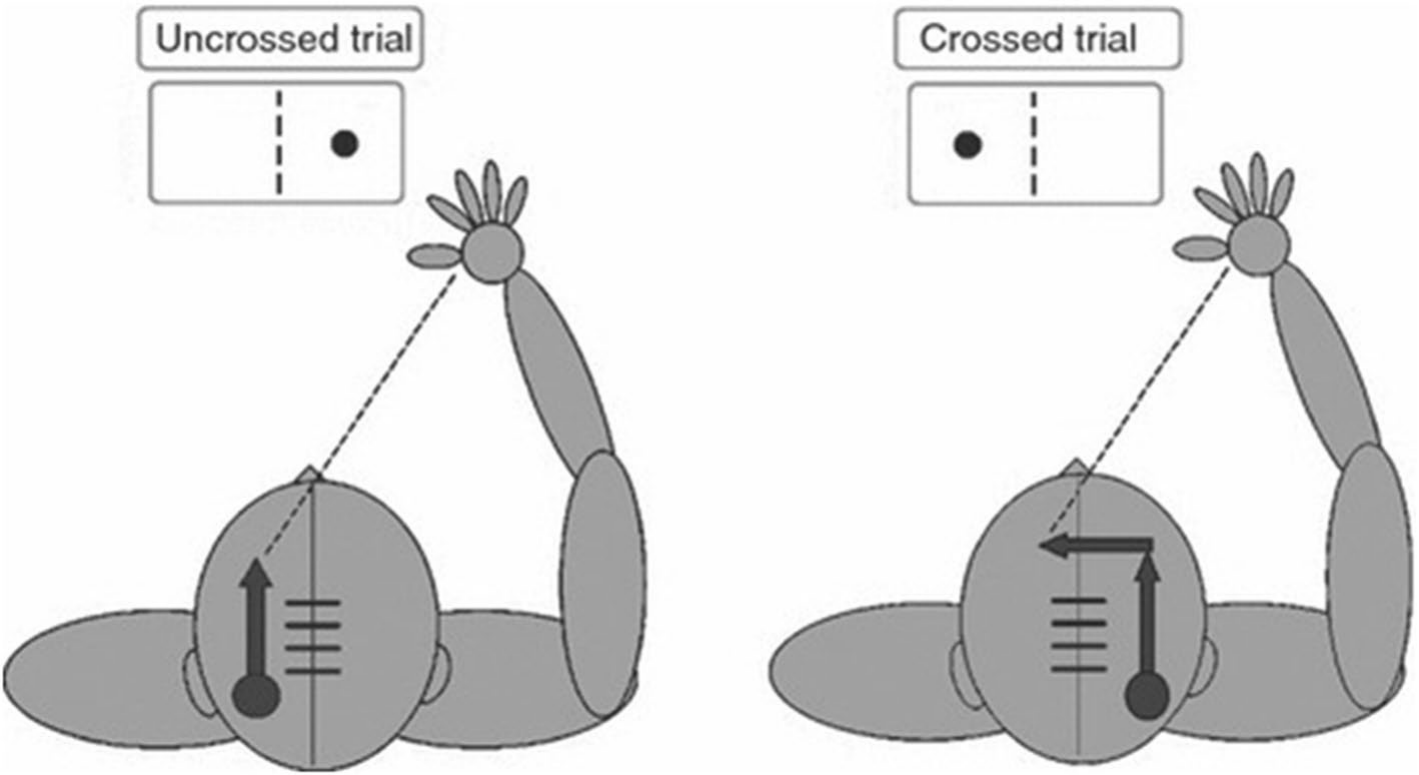
Poffenberger’s behavioral paradigm is meant to estimate interhemispheric delay, since the crossed trial requires information to cross the CC. Available from: https://www.researchgate.net/figure/224924768. Notice that a somatosensory variant of the same task also exists (Tamè and Longo 2015)

Below, I will summarize the long struggle to understand the NONs underlying an apparently simple behavior in the human brain, the so-called Poffenberger’s paradigm (Fig. 6).

Albert Poffenberger (1885–1974; Devonis 2012) belonged to a family of physicians, did his undergraduate studies at Bucknell, and in 1909 went to Columbia for postgraduate studies. For his thesis work, he implemented a rather unique attempt to measure the temporal properties of a CNS circuit by psychological methods. Since each hemisphere has access to only one-half of the visual field, and controls only half of the body musculature, it should be possible to assess the delay introduced by the task of responding with one hand to stimuli presented to the visual hemifield of the other hemisphere (the crossed condition) vs the delay of the response when the stimulus is presented to the hemifield of the hemisphere controlling the hand (the uncrossed condition). The difference between those two delays is much greater when the corpus callosum is absent or sectioned, consistent with the hypothesis that the task measures the delay generated by interhemispheric communication, possibly between cortical areas of the two hemispheres. One problem with this interpretation is that the delays returned by Poffenberger’s paradigm are short (3–5 ms) both when measured in the visual and in the somatosensory modality (Tamè and Longo 2015). Unfortunately, the computed delays of the interhemispheric connections between prefrontal and motor areas (10 ms) or parietal areas (12–13 ms) are longer than the behavioral estimates while those between visual areas are even longer. One possible explanation of the discrepancy is that the behavior is mediated by the fastest interhemispheric axons, that is, results from a race between axons of different conduction velocities (Marzi 2010).

To clarify which areas are involved in the task, two groups provided fMRI evidence, by subtracting the cortical activation during the uncrossed condition from the crossed condition. In the crossed condition, Tettamanti et al. (2002) reported a complex picture including activation of premotor cortex (BA 6), mesial prefrontal cortex (BA 9), and paracentral lobule (BA 5). Interestingly, the bold signal also increased in the genual part of CC, which contain prefrontal as well as premotor axons suggesting that these axons were also activated. In a comparable study, Iacoboni and Zaidel (2004) reported activation in the prefrontal premotor and superior parietal areas. Weber et al. (2005) repeated the fMRI study with the intent of teasing apart areas involved in interhemispheric transmission from those concerned with spatial attention. They confirmed the involvement of the superior parietal cortex (BA 7), premotor cortex (BA 6), and prefrontal cortex (BA 9/10) as well as activation of the fibers in the genu of CC.

**Fig. 7:**
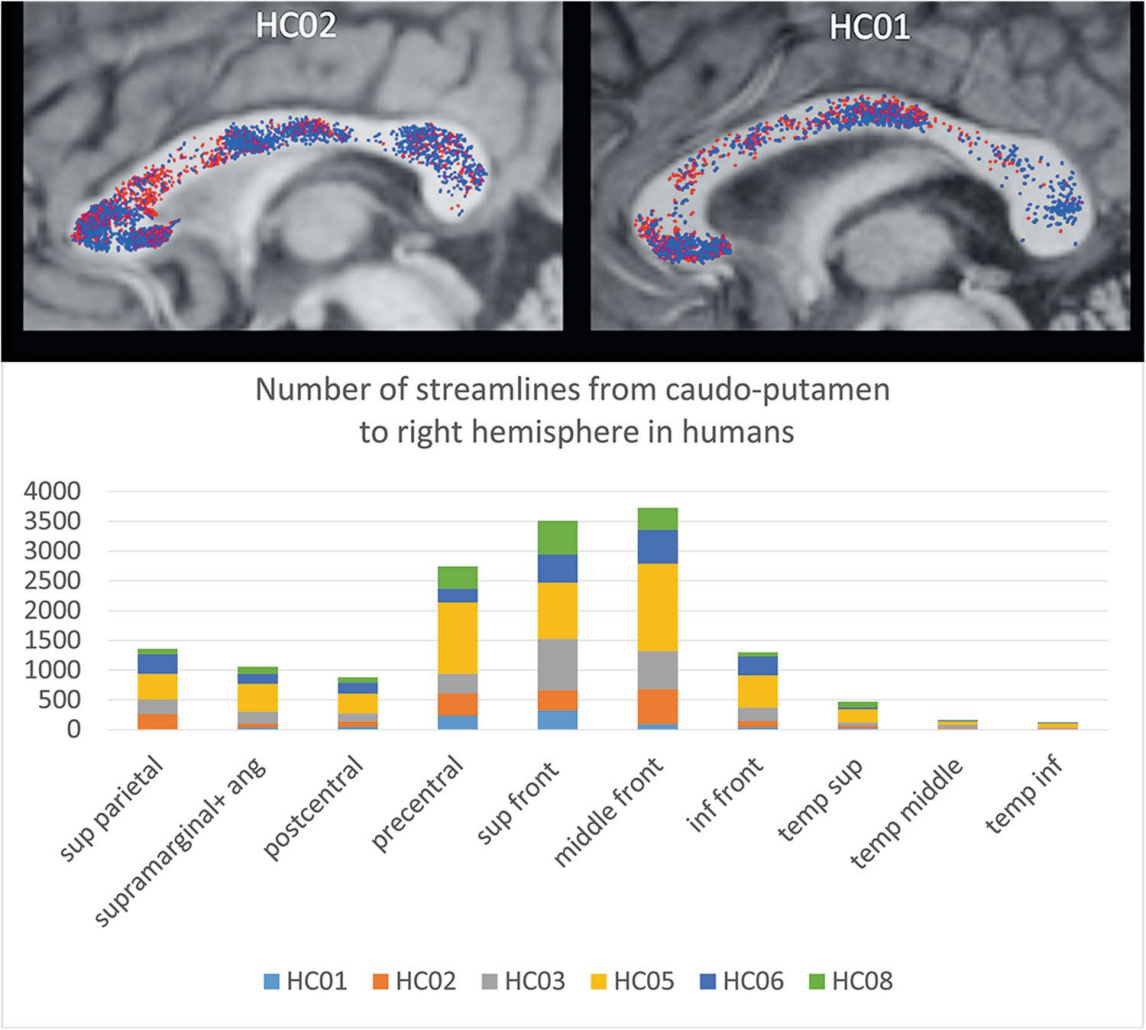
*Upper panel* streamlines connecting left caudate (*red*) and left putamen, through the CC, to the contralateral hemisphere in two human cases. *Lower panel* the cortical areas to which streamlines were traced in human cases. Notice a peak in prefrontal, and precentral areas, and a substantial contribution to superior parietal areas, in spite of individual variability. (Modified from Innocenti et al. 2016b)

An interhemispheric cortical projection to the contralateral striatum was recently re-visited in monkeys and humans using a combination of tract tracing (in monkeys) and of diffusion tractography (DT) in both (Innocenti et al. 2016b). The areas preferentially activated in the crossed–uncrossed conditions in Poffenberger’s paradigm, namely, the prefrontal, and premotor areas send projections to the contralateral striatum, via in the corpus callosum, in both monkey and humans (Fig. 7). In both species, the axons cross in the anterior CC, consistent with the preferential activation detected by fMRI (above). In addition, in humans, but not in monkey, a crossed projection to the striatum originates from superior parietal cortex, and crosses in the isthmus of CC. Interestingly, a permanent elongation of the crossed-uncrossed delay was observed in one patient with CC damage involving the isthmus of CC (but probably also the mid-body; Peru et al. 2003).

The crossed cortico-striatal projection is bound to be faster than the cortico-cortical-subcortical route for some tasks, notably the repetitive, automated motor tasks required by Poffenberger’s paradigm. Indeed, in the monkey, the delays calculated from prefrontal cortex are 3.4 ms for the ipsilateral and 5.1 ms for the contralateral projection to n caudatus (Innocenti et al. 2016b). By taking into account, an increase of axon diameters in humans in the order of 30% (compatible with macaque/human comparative data) and of conduction distance proportional to the cubic route of brain volumes, validated by DT tracing, the ipsi- and contra-calculated values in humans is 6.6 and 9.9 ms. This means an average difference of 3.3 ms between the ipsilateral and the contralateral projection, which corresponds rather precisely to the differences returned by the behavioral paradigm. Thus, the Poffenberger’s task might involve crossed cortico-striatal, rather than cortico-cortical connections through the CC (Innocenti et al. 2016b).

## Conclusions

The alleged identification of the pathways involved in a simple task such as Poffenberger’s paradigm, even if it were found to be correct, provides a small consolation to the hope that one might precisely relate brain circuits to function. Indeed, even for such a simple task, the full definition of the NONs involved is far from exhaustive. Questions include (1) what is the involvement of the different areas identified by fMRI analysis in the task? (2) Do the projections they send out cooperate or compete? (3) Are the areas involved in the interhemispheric transfer or rather in associated intrahemispheric computations? (4) Which parts of the striatum are involved in the circuits mediating behavior and how? (5) The analysis of single axons to the striatum suggests that each of them distributes rather diffusely through caudo-putamen requiring summation with other inputs to fire the target neurons (Innocenti et al. 2016b). Which type of information do they carry? (6) Finally, what is the structure of the NONs driven through striatum?

These questions are, in principle, answerable, but they stress the considerable amount of anatomical and functional knowledge required to describing behavior and cognition in terms of causal NONs.

## References

[CR1] Aboitiz F, Scheibel a B, Fisher RS, Zaidel E (1992). Fiber composition of the human corpus callosum. Brain Res.

[CR2] Anderson JC, Binzegger T, Douglas RJ, Martin KAC (2002). Chance or design? Some specific considerations concerning synaptic boutons in cat visual cortex. J Neurocytol.

[CR3] Branco T, Häusser M (2011). Synaptic integration gradients in single cortical pyramidal cell dendrites. Neuron.

[CR4] Caminiti R, Ghaziri H, Galuske R (2009). Evolution amplified processing with temporally dispersed slow neuronal connectivity in primates. Proc Natl Acad Sci USA.

[CR5] Debanne D, Campanac E, Bialowas A (2011). Axon physiology. Physiol Rev.

[CR6] Devonis D C (2012) Poffenberger AT. In: Rieber RW (ed) Encyclopedia of the history of psychological theories. doi:10.1007/978-1-4419-0463-8, Springer, LLC

[CR7] Fromm C, Evarts EV (1981). Relation of size and activity of motor cortex pyramidal tract neurons during skilled movements in the monkey. J Neurosci.

[CR8] Grillner S, Kozlov A, Dario P (2007). Modeling a vertebrate motor system: pattern generation, steering and control of body orientation. Prog Brain Res.

[CR9] Iacoboni M, Zaidel E (2004). Interhemispheric visuo-motor integration in humans: the role of the superior parietal cortex. Neuropsychologia.

[CR10] Ibrahim M, Butt AM, Berry M (1995). Relationship between myelin sheath diameter and internodal length in axons of the anterior medullary velum of the adult rat. J Neurol Sci.

[CR11] Innocenti GM, Finlay B, Innocenti GM, Scheich H (1991). Pathways between development and evolution. The Neocortex.

[CR12] Innocenti GM (1993) Organisation de l’ecorce cerebrale et “projet” des neurosciences. In Ansermet F, Innocenti GM, Steck A (eds) Payot, Lausanne, pp 35–44

[CR13] Innocenti GM (2011). Development and evolution: two determinants of cortical connectivity. Prog Brain Res.

[CR14] Innocenti GM, Kaas JH (2017). Evolutionary-developmental aspect of cortical connectivity. Evolution of nervous systems.

[CR15] Innocenti GM, Caminiti R (2016) Axon diameter relates to synaptic bouton size: structural properties define computationally different types of cortical connections in primates. Brain Struct Funct. doi:10.1007/s00429-016-1266-1 10.1007/s00429-016-1266-1 27372337

[CR16] Innocenti GM, Vercelli A, Caminiti R (2014). The diameter of cortical axons depends both on the area of origin and target. Cereb Cortex.

[CR17] Innocenti GM, Carlén M, Dyrby TB (2016a) Chap. 15—the diameters of cortical axons and their relevance to neural computing. Axons Brain Archit 317–335. doi:10.1016/B978-0-12-801393-9.00015-3

[CR18] Innocenti GM, Dyrby TB, Andersen KW et al (2016b) The crossed projection to the striatum in two species of monkey and in humans: behavioral and evolutionary significance. Cereb Cortex bhw161. doi:10.1093/cercor/bhw161 10.1093/cercor/bhw161 27282154

[CR19] Jiang X, Shen S, Cadwell CR et al (2016) Principles of connectivity among morphologically defined cell types in adult neocortex. doi:10.1126/science.aac9462 10.1126/science.aac9462 PMC4809866 26612957

[CR20] Lamantia a S, Rakic P (1990). Cytological and quantitative characteristics of four cerebral commissures in the rhesus monkey. J Comp Neurol.

[CR21] Makarov Va, Schmidt KE, Castellanos NP (2008). Stimulus-dependent interaction between the visual areas 17 and 18 of the 2 hemispheres of the ferret (*Mustela putorius*). Cereb Cortex.

[CR22] Markram H, Muller E, Ramaswamy S (2015). Reconstruction and simulation of neocortical reconstruction and simulation of neocortical microcircuitry. Cell.

[CR23] Marr D (1982). Vision. A computational investigation into the human representation and processing of visual information.

[CR24] Marzi CA (2010) Asymmetry of interhemispheric communication. Wiley Interdiscip Rev Cogn Sci 1:433–438. doi:10.1002/wcs.53 10.1002/wcs.53 26271383

[CR25] Massé I O, Régnier P, Boire D (2016) Chap. 5. Geometrical structure of single axons of visual cortica connections in the mouse. Axons Brain Archit 93–116. doi:10.1016/B978-0-12-801393-9.0005-0

[CR26] Mengual E, Prensa L, Tripathi A et al (2016) Chap. 3. Comparative analysis of the axonal collateralization patters of basal ganglia output nuclei in the rat. Axons Brain Archit 47–68. doi:10.1016/B978-0-12-801393-9.0003-7

[CR27] Parent M, Parent A (2016) Chap. 2. The primate basal ganglia connetome as revealed by single-axon tracing. Axons Brain Archit 27–46. doi:10.1016/B978-0-12-801393-9.0002-5

[CR28] Paxinos G, Xu-Feng H, Toga WT (2000). The rhesus monkey brain.

[CR29] Perge JA, Niven JE, Mugnaini E (2012). Why do axons differ in caliber?. J Neurosci.

[CR30] Peru A, Beltramello A, Moro V (2003). Temporary and permanent signs of interhemispheric disconnection after traumatic brain injury. Neuropsychologia.

[CR31] Tamè L, Longo MR (2015) Inter-hemispheric integration of tactile-motor responses across body parts. Front Hum Neurosci 9:1–8. doi:10.3389/fnhum.2015.00345 10.3389/fnhum.2015.00345 PMC4466437 26124718

[CR32] Tettamanti M, Paulesu E, Scifo P et al (2002) Interhemispheric transmission of visuomotor information in humans: fMRI evidence. J Neurophysiol 88:1051–1058. doi:10.1152/jn.00417.2001 10.1152/jn.2002.88.2.1051 12163553

[CR33] Tettoni L, Gheorghita-Baechler F, Bressoud R (1998). Constant and variable aspects of axonal phenotype in cerebral cortex. Cereb Cortex.

[CR34] Tomasi S, Caminiti R, Innocenti GM (2012). Areal differences in diameter and length of corticofugal projections. Cereb Cortex.

[CR35] Weber B, Treyer V, Oberholzer N (2005). Attention and interhemispheric transfer: a behavioral and fMRI study. J Cogn Neurosci.

